# Improving understanding of the functional diversity of fisheries by exploring the influence of global catch reconstruction

**DOI:** 10.1038/s41598-017-10723-1

**Published:** 2017-09-06

**Authors:** Kirsty L. Nash, Reg A. Watson, Benjamin S. Halpern, Elizabeth A. Fulton, Julia L. Blanchard

**Affiliations:** 1 Centre for Marine Socioecology, Private Bag 129, Hobart, TAS 7001 Australia; 2 Institute for Marine and Antarctic Studies, University of Tasmania, Private Bag 129, Hobart, TAS 7001 Australia; 3 National Centre for Ecological Analysis and Synthesis, University of California, 735 State St, Santa Barbara, CA 93101-5504 USA; 4 Bren School of Environmental Science & Management, University of California, Santa Barbara, CA 93101 USA; 5 Imperial College London, Silwood Park Campus, Burkhurst Rd., Ascot, SL57PY UK; 6 CSIRO, Oceans and Atmosphere, Castray Esplanade, Battery Point, TAS 7004 Australia

## Abstract

Functional diversity is thought to enhance ecosystem resilience, driving research focused on trends in the functional composition of fisheries, most recently with new reconstructions of global catch data. However, there is currently little understanding of how accounting for unreported catches (e.g. small-scale and illegal fisheries, bycatch and discards) influences functional diversity trends in global fisheries. We explored how diversity estimates varied among reported and unreported components of catch in 2010, and found these components had distinct functional fingerprints. Incorporating unreported catches had little impact on global-scale functional diversity patterns. However, at smaller, management-relevant scales, the effects of incorporating unreported catches were large (changes in functional diversity of up to 46%). Our results suggest there is greater uncertainty about the risks to ecosystem integrity and resilience from current fishing patterns than previously recognized. We provide recommendations and suggest a research agenda to improve future assessments of functional diversity of global fisheries.

## Introduction

The functional roles played by individual species within a community, such as herbivory, pollination and nitrogen fixation, and the diversity of these roles, directly support key processes that underpin ecosystem structure, dynamics and resilience^1^. Thus, the functional composition of a community will influence the capacity of an ecosystem to recover following disturbance, and the delivery of ecosystem services on which human societies rely^2, 3^. As a result, there are increasing efforts to characterise spatio-temporal patterns in functional composition of species from local to global scales^2, 4^. This research has traditionally used qualitative functional groupings such as ‘herbivore’ to explore functional diversity, but more recent studies have been based on a series of categorical and continuous traits such as size and depth range, that quantitatively describe the functional role of a species or individual by defining its position in a multi-dimensional trait space^5^.

Most global-scale functional diversity work has focused on terrestrial systems^6^, or specific marine ecosystems such as coral and rocky reefs^7, 8^. However, an emerging literature exploring functional patterns using global fisheries datasets is supporting broad-scale investigation of functional trends through time and across space^9^. In particular, information arising from these fisheries studies is being used to assess progress towards marine aspects of the Convention on Biological Diversity and thus has the potential to influence environmental policy^10, 11^.

**Figure 1:**
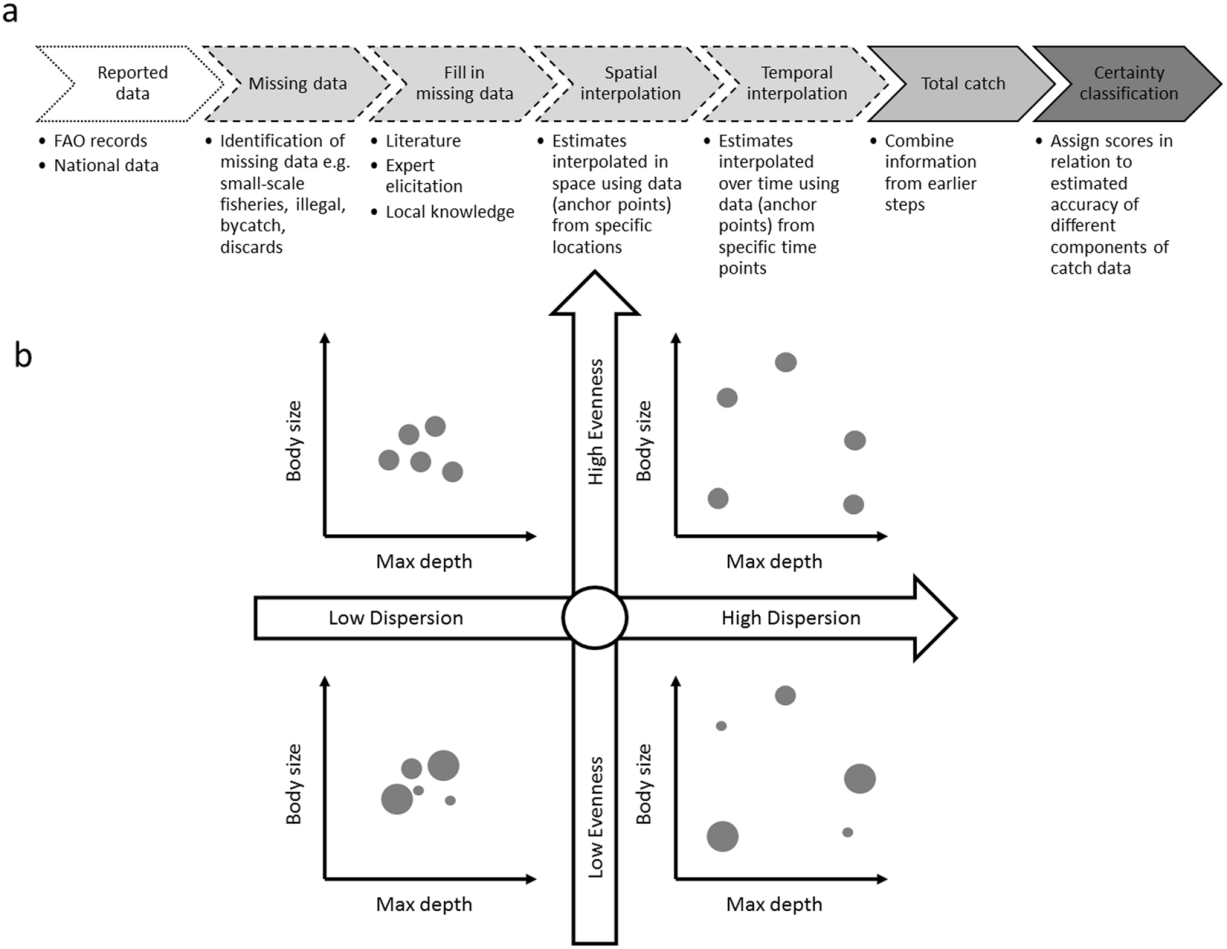
Catch reconstruction process and functional diversity metrics. (**a**) Sources and flow of information used in catch reconstruction process from the reported data, addition of unreported data (including small-scale and illegal fisheries, bycatch and discards), and classification of estimated certainty of catch data. Certainty scores are based on qualitative expert assessment of the relative accuracy of the catch estimate derived from the underlying data, assumptions and methods used for each sector and country, and were developed from a similar approach used in the IPCC process. (**b**) Four examples of catch data displayed in trait space, with tonnage caught of each taxon indicated by bubble size. Panels illustrate differences between communities with the same number of taxa and total biomass but varying levels of dispersion and evenness. For illustrative purposes, the two traits body size and maximum depth are shown. Figure (A) created from information in ref. ^13^

Long-term (1950–2010) data made freely available by the Sea Around Us project (SAUP) provide an invaluable record of the composition and tonnage of fisheries catches for Exclusive Economic Zones (EEZ) of the world^12^. SAUP catch data are reconstructed from Food and Agricultural Organization (FAO) fisheries records of reported landings from each country. Reported data are supplemented by country-level estimates of unreported catch using literature searches and expert elicitation (Fig. 1a) following methodology provided by^13^, but carried out by a range of country specific research teams. Certainty scores are assigned to reconstructed data to indicate qualitative expert assessment of the relative accuracy of the catch estimate derived from the underlying data, assumptions and methods used for each sector and country^13^. The addition of estimated unreported catch (e.g., small-scale and illegal fisheries, bycatch, and discards) has proved vital in providing a more complete picture of fisheries, for example, highlighting a considerably greater global magnitude (annual peak of 86 vs 130 million tonnes) and contrasting trajectory of fish extraction from the oceans than indicated by the FAO database^14^.

A range of studies are now using these data to explore how fisheries select for specific traits that underpin the functional role of fish species, such as size and trophic level^15^, and how this selection may vary in time and space due to shifts in fishing behaviour^16, 17^. Global fisheries data also improve our understanding of how changing environmental conditions, e.g. climate, may drive shifts in the catch of species with specific functional traits^18^, and how functional diversity may buffer the negative impacts of temperature variability on fisheries productivity^9^. Thus, trends in functional diversity of catches reflect fishing selectivity, changes in fishing behaviour and technologies, and environmental change^18–21^. Teasing apart the differential effects of these drivers is an important research challenge necessary to inform management of different human activities and impacts on community and ecosystem resilience. Our ability to address this challenge is dependent on a clear understanding of functional diversity trends in both reported and unreported components of the catch. Adding unreported data provides a different picture of the magnitude of fisheries exploitation, indicating a peak in catch 39% (uncertainty band from −15 to 90%) greater than previously thought^14^. But to date there has been no exploration of the impact of unreported catches, such as the often overlooked multi-species subsistence and artisanal fisheries (hereafter termed ‘small-scale’)^22^, on broad-scale functional trends. This knowledge is critical because it explores the need for robust reconstruction of underreported elements of global fisheries to understand functional trends and support ecosystem-based fisheries management.

Here, we investigated how the addition of estimated unreported catches to global fisheries data influence spatial patterns in functional diversity for a single year, 2010. First, we evaluate functional evenness in catch, which indicates how evenly catches are distributed in trait space and thus the relative dominance of certain trait combinations or functions. Second, we evaluate functional dispersion, which indicates how catches are spread out in trait space and thus the breadth of different trait combinations or functions (Fig. 1b). Specifically, we asked: (1) How do functional estimates vary among different groupings within the catch – reported vs. unreported, small-scale vs. industrial fishing, and low and high certainty data? (2) How does the addition of these different groupings influence spatial patterns in functional diversity of the total catch? (3) What is the impact of using either coarse functional groups or finer-scale trait information on the above patterns of functional diversity?

Unreported and reported data, and small-scale and industrial fisheries focus on distinct parts of the catch, thus, we hypothesised that these catch groupings would exhibit different functional diversity values within EEZs. Due to large variability in fishing practices and habitats among EEZs^23, 24^, we hypothesised that there was unlikely to be coherent broad-scale distinctions in the functional fingerprints of these catch groupings across different EEZs. Nonetheless, as unreported elements incorporate different sectors, gears and fishing practices, overall, at the global scale, accounting for unreported elements would likely influence functional diversity patterns. Functional groups lack the resolution of trait-based analyses, as such we hypothesised that functional diversity patterns estimated from functional groups would show less clear functional differences among catch groupings, than patterns derived from trait data.

## Results

### Spatial variation in functional diversity

**Figure 2:**
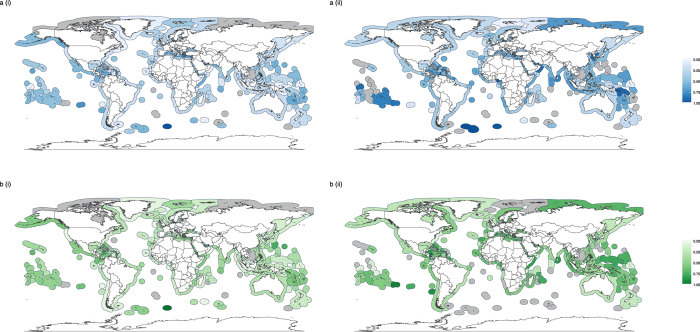
Spatial variation in functional evenness. Functional evenness in (**ai**) reported, (**aii**) unreported, (**bi**) industrial and (**bii**) small-scale catches for EEZs in 2010. Estimation of functional evenness based on fine-scale trait data and catch identified to species or genus level. Grey shading in EEZs represents missing data, where there were either too few functionally distinct taxa to estimate evenness or insufficient data at the species/genus level. Maps created in using the ggplot2^45^ package in R^46^.

**Figure 3:**
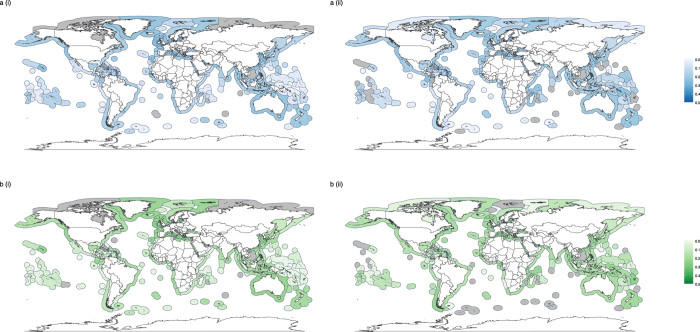
Spatial variation in functional dispersion. Functional evenness in (**ai**) reported, (**aii**) unreported, (**bi**) industrial and (**bii**) small-scale catches for EEZs in 2010. Estimation of functional dispersion based on fine-scale trait data and catch identified to species or genus level. Grey shading in EEZs represents missing data, where there were either too few functionally distinct taxa to estimate dispersion or insufficient data at the species/genus level. Maps created in using the ggplot2^45^ package in R^46^.

There was geographic variation among EEZs in the functional evenness (Fig. 2 & Supplementary Fig. S1) and dispersion (Fig. 3 & Supplementary Fig. S2) of different catch groupings. However, there do not appear to be clear latitudinal or ocean-specific patterns in either functional evenness or functional dispersion. Furthermore, those EEZs reporting a greater proportion of their catch or with a greater proportion of industrial catch did not exhibit consistently higher or consistently lower functional evenness or dispersion (Supplementary Note 1). It should be noted that there were strong correlations among the proportion of reported catch and the proportion of the catch arising from industrial fishing (Supplementary Note 2). EEZs with the greatest functional evenness based on fine-scale trait data did not necessarily have the highest evenness values when based on coarse functional groups for any catch groupings (dark shading in Fig. 2 vs Supplementary Fig. S1). Due to the large amount of missing data on certainty classifications (Supplementary Note 2), trends arising from data on the certainty classifications are likely to be misleading. As a result, certainty classifications were not used in the functional diversity analyses.

### Functional fingerprint of catch groupings

**Figure 4:**
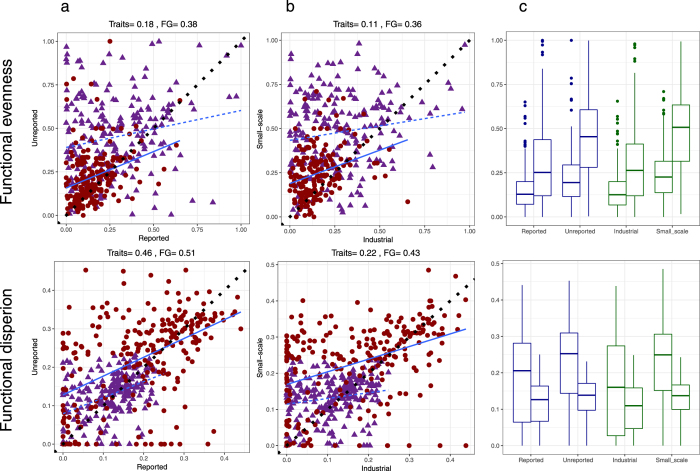
Relationships between functional metrics for different catch groupings. Correlations among estimates of functional metrics for (**a**) unreported vs reported data and (**b**) small-scale (subsistence and artisanal) vs industrial fisheries. (**c**) Range of values for different catch groupings. In (**a**) and (**b**) dotted black line is y = x relationship and statistics presented are spearman rank rho values. Triangles, dashed lines and purple shading are estimates using trait information, circles, solid lines and red shading are estimates using broad functional groups (FG).In (**c**) in each pair of boxplots, first boxplot represents estimates using broad functional groups and second boxplot represents estimates using trait information.  Each data point is an EEZ in 2010.

Functional estimates for reported and unreported, and industrial and small-scale fisheries were only weakly to moderately positively correlated, as hypothesised (Fig. 4a,b; Spearman rank rho = 0.11 to 0.51). Both functional evenness and functional dispersion were significantly greater in unreported and small-scale fishery catch components compared to reported and industrial catch components for EEZs in 2010 (Table 1; Fig. 4c). This conflicted with our expectations that broad-scale trends in functional fingerprints would be masked by large variation in habitat and fishing practices among EEZs. These results hold whether estimates were calculated using either fine-scale traits or coarse functional groups (Table 1; Fig. 4c).

**Table 1:** Wilcoxon-paired tests comparing median values of functional metrics: A) functional evenness, and B) functional dispersion, for unreported vs. reported data and small-scale (subsistence and artisanal) vs. industrial fisheries for EEZs in 2010, and when using fine-scale trait data or broad functional groups to estimate metrics.

	Traits	Functional groups
**A) Functional Evenness**		
Reported vs. Unreported	Z = 4491; P < 0.001	Z = 5392; P < 0.001
Industrial vs. Small-scale	Z = 3073; P < 0.001	Z = 2721; P < 0.001
**B) Functional Dispersion**		
Reported vs. Unreported	Z = 10653; P = 0.007	Z = 9045; P < 0.001
Industrial vs. Small-scale	Z = 8319; P < 0.001	Z = 5837; P < 0.001

### Influence of functional fingerprint of catch groupings on patterns in total catch

**Figure 5:**
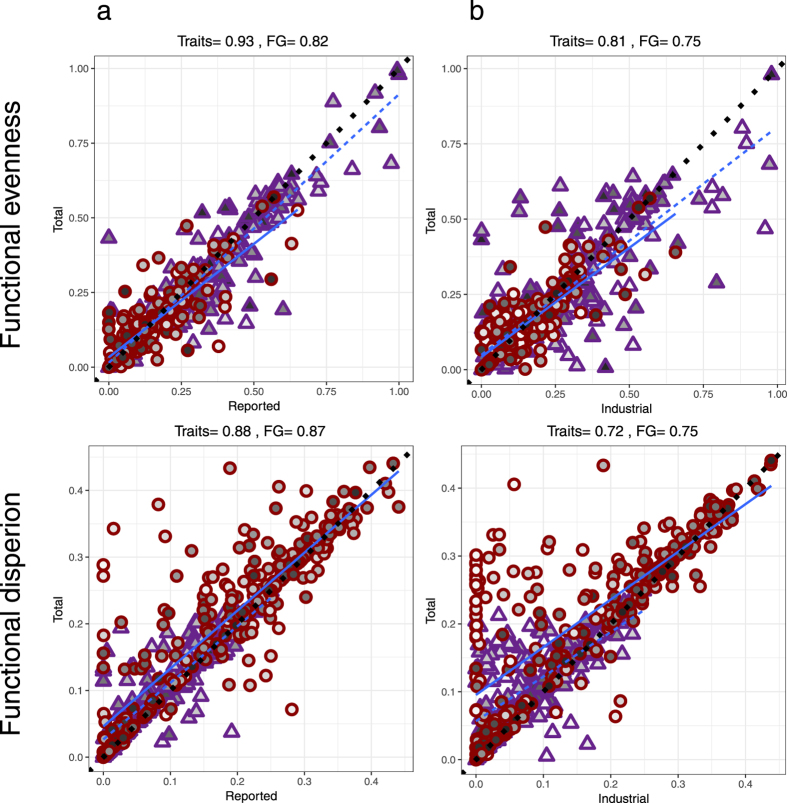
Correlations among estimates of functional metrics for total catch versus different catch groupings. (**a**) Reported data and (**b**) industrial catch. Dotted black line is y = x relationship and statistics presented are spearman rank rho values. Each data point is an EEZ in 2010. Purple triangles and dashed lines are estimates using trait information, red circles and solid lines are estimates using broad functional groups (FG). Shading represents proportion of the catch that is either reported or industrial (low values-light shading, high values-dark shading).

Despite the differences in functional estimates for unreported versus reported data and small-scale versus industrial catches, there were strong positive correlations between functional estimates for the total catch, and both reported and industrial sectors (Spearman rank rho = 0.672–0.93, Fig. 5). Most EEZs were comprised primarily of reported and industrial data (Supplementary Fig. S7); consequently, adding unreported and small-scale catches to the database has little impact on functional diversity estimates across EEZs. Those EEZs showing greatest differences in functional estimates between the total catch and either reported or industrial catches were often, but not always, characterised by a smaller proportion of reported or industrial data within their total catches (highlighted by shading of symbols in Fig. 5).

### Spatial variation in influence of functional fingerprints on patterns in total catch

At the global scale, accounting for unreported data and small-scale fisheries appear to have little impact on functional diversity estimated for the total catch. However, there is geographic variation in the proportions of catch within these groupings among EEZs (Supplementary Figs S3 & S4). This variation in proportion of data within different catch groupings highlights the extent of reconstruction required in each EEZ, and the relative potential influence of the functional fingerprint of different groupings on trends in the total catch.

**Figure 6:**
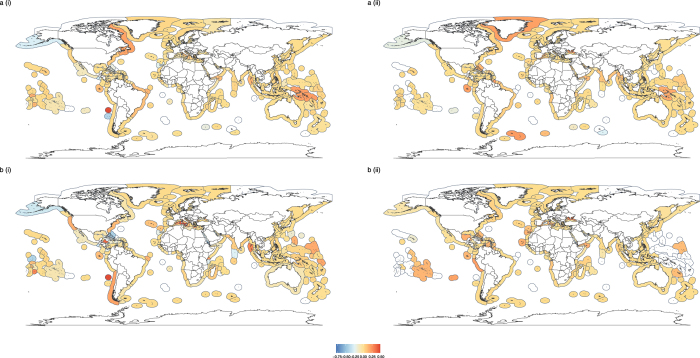
Difference between functional evenness estimates of total catch data and (**a**) reported and (**b**) industrial data, where metrics are estimated using either (i) detailed trait information or (ii) broad functional groups for EEZs in 2010. White shading in EEZs represents missing data, where there were either too few functionally distinct taxa to estimate evenness or insufficient data at the species/genus level (trait analysis only). Maps created in using the ggplot2^45^ package in R^46^.

**Figure 7:**
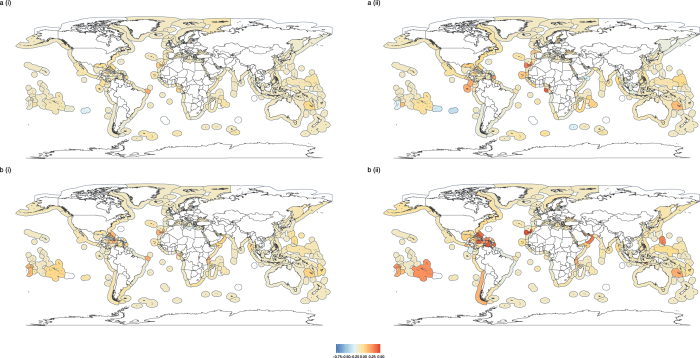
Difference between functional dispersion estimates of total catch data and (**a**) reported data and (**b**) industrial data, where metrics are estimated using either (i) detailed trait information or (ii) broad functional groups for EEZs in 2010. White shading in EEZs represents missing data, where there were either too few functionally distinct taxa to estimate dispersion or insufficient data at the species/genus level (trait analysis only). Maps created in using the ggplot2^45^ package in R^46^.

Global maps of functional metrics show differences between total catch functional metric estimates and those for reported and industrial fishing, and were used to highlight specific EEZs where accounting for functional estimates of small-scale and unreported data may be particularly important (Figs 6 and 7). Once again, there did not appear to be any clear latitudinal or ocean-specific patterns in the magnitude of differences between functional estimates of the total catch and of either reported or industrial catches. Many EEZs showed little difference in functional evenness estimates between the total catch and specific groupings within the catch (yellow shading in Fig. 6). However, some EEZs showed larger differences, even where a high proportion of catch was reported or from the industrial sector. For example, lower functional evenness was observed in reported and industrial catches compared to the total catch (positive difference values; orange/red shading in Fig. 6) in the trait based analysis for the Desventuradas Islands of Chile (Fig. 6a(i); 70% catch reported & b (i); 99% industrial catch). A similar trend was found for reported catches in the functional group based analysis for Greenland (Fig. 6a(ii) 94% catch reported). In contrast, the functional evenness estimated from reported and industrial data was greater than for the total catch (negative difference values; blue shading in Fig. 6) in the trait based analyses for subarctic Alaska (Fig. 5a(i); 97% catch reported & b(i); 76% industrial catch).

In general, the difference in functional dispersion estimates between the total catch and different groupings was lower than for functional evenness, and many EEZs that showed large differences in functional evenness between the total catch and specific groupings did not show large differences in functional dispersion (EEZs with paler shading in Fig. 7 than in Fig. 6).

## Discussion

Information arising from studies of spatial and temporal patterns in fisheries functional traits and diversity is important for understanding the delivery and future vulnerability of ecosystem services, and is currently being used to assess progress towards attainment of the Aichi biodiversity targets^10, 11^. Thus, exploring how reporting of fisheries influences our understanding of broad-scale and regional trends in function is critical for ensuring robust data are available to support environmental policy. Accounting for unreported catches provides a very different picture of the total amount of fish removed from the world’s oceans than provided by reported data alone^14^. Interestingly, our findings suggest that at a global scale, accounting for unreported catches has little impact on patterns in functional diversity. However, this large-scale outcome masks regional variation and locations where accounting for unreported catch is essential for understanding trends in functional diversity. Critically, it is at these local to regional scales that nearly all fisheries and ecosystem management decisions are made, suggesting that in order to effectively inform on-the-ground management, studies of functional diversity trends should account for unreported components of the catch.

The incredible diversity of environmental conditions, habitats and fishing practices among EEZs^23, 24^ will affect the traits and functional diversity of fish caught^9, 16, 18, 20^. Despite this considerable variability in drivers of community composition, it is still possible to discern broad-scale differences in the functional fingerprints of reported and unreported, and industrial and small-scale components of fisheries: unreported and small-scale catches tend to be more functionally even and more functionally dispersed than reported and industrial catches. This finding contrasts with our initial hypothesis, and suggests that there may be more consistent functional characteristics of unreported data across EEZs than anticipated. Critically, this outcome suggests that where little information is available on the composition of unreported catches, and where uncertainty surrounding the reconstruction process is high, coarse qualitative estimates of functional diversity in unreported components may still be possible.

Despite differences in functional diversity among catch groupings, the impact of unreported and small-scale fisheries data on functional estimates for the total catch are relatively small at the global scale. This finding conflicted with our initial hypothesis, and appears to be driven, in part, by the high proportion of reported and industrial data within the total catch. Thus, at the global scale, removing unreported and small-scale catch does not have a large influence on functional estimates. However, the proportion of catch that is reported varies over time^14^ and differs among EEZ’s. For example, the Cocos Keeling Islands have seen a decrease in the percentage of catch reported from 67% to 0% between 1950 and 2010, whereas the Barents Sea (Russia) has seen an increase from 39% to 73% over the same period. Furthermore, at the EEZ scale, the effect of accounting for unreported catches had a considerable impact on functional diversity estimates in some locations. Both the spatio-temporal variability in reporting of data, and the regional scale impacts of unreported data on functional estimates suggests that it is critical to account for unreported components of the catch. Specifically, including unreported catches, such as small-scale fisheries, will help clarify temporal trends in functional diversity, improve the precision of estimates at global scales and help quantify human impacts on diversity at local to regional scales^2, 4, 25, 26^. Such efforts are critical to inform effective fisheries management focused on supporting ecosystem function.

Coarse-grained functional groupings provide a simple and tractable approach to classifying the roles species play. However, this approach may miss subtle, yet potentially important, distinctions among individuals and species^27^. As a result, there have been moves to use detailed characterisations of species’ functions based on life-history and ecological traits, giving a more nuanced appreciation of functional roles^5, 28^. Although, there were differences in the functional diversity estimates between the analyses based on functional group and trait data, the overall findings of our study were robust to the type of information used to describe function. These results indicate that even when using relatively coarse-grained functional group information it is possible to see differences in the functional fingerprints of reported and unreported, industrial and small-scale components of the catch. This finding contrasts with our initial hypothesis, and suggests that functional groupings already available within the SAUP database may be used to explore research questions related to broad-scale trends in functional diversity. The advantage of this approach is that it allows usage of the full catch dataset. Functional groups are available for both fish and invertebrate species, unlike trait information that is very limited for certain taxa within the catch, such as invertebrates, and may only be used with any precision for taxa identified to species or genus level. Nonetheless, where studies are focused on trends in particular traits, such as changing trophic level^16^ or temperature tolerance^9^, rather than functional diversity, more detailed trait information may be appropriate. In this context, enrichment of existing trait information for both fish and invertebrates is critical (Recommendation 1, Table 2). Furthermore, selection of specific traits to incorporate into functional analyses will depend on the research question. Sensitivity analyses commonly used in trait and functional diversity studies, such as assessing the impact of including specific traits and the degree of pooling within trait categories^7, 9, 16^ are necessary to ensure optimal trait selection and definition.

**Table 2:** Recommendations related to data quality and methodologies to improve the robustness of functional analyses based on global fisheries datasets.

Stage	Recommendation
Functional analysis	1. Data: Highlight patterns in trait coverage, such as variation in space. Enrich trait information available from FishBase where possible with other databases such as the Ocean Biogeographic Information System (http://www.iobis.org/) and the Global Biodiversity Information Facility (http://www.gbif.org/).
	2. Methods: Careful choice of functional metric. In EEZs where there is high proportion of unreported data and low confidence in catch reconstruction, functional dispersion may be less affected by missing data than functional evenness.
	3. Methods: Comparison of patterns in functional diversity using fisheries-dependent and independent data to understand the links between exploitation patterns and ecosystem function.
Certainty	4. Data: Increased comprehensiveness of certainty data in catch database.
	5. Methods: Assessment of differences in functional diversity estimates among low and high certainty data.
Reconstruction of missing data	6. Data: For each EEZ more detail is needed in background documents relating to each step of the reconstruction process, such as data sources used and assumptions made.
	7. Data: Need to enrich data with fleet attributes such as gear type so can assess selectivity effects on functional diversity.
	8. Methods: Simulation testing exploring the effect of different reconstruction assumptions and approaches versus gear selectivity effects on functional diversity estimates.
Reporting	9. Data: Improved taxonomic resolution of FAO catch reporting is needed.
	10. Methods: Assessment of the impact of temporal changes in taxonomic resolution in FAO dataset on functional diversity estimates.

Shading corresponds to Fig. 1a, black shading represents steps beyond those shown in Fig. 1a.

Understanding the causes and consequences of these changes in functional diversity depends, in part, on how functional diversity is measured. Different functional metrics provide distinct and often complementary perspectives on trends in functional diversity, therefore, metric choice should be tailored to the research aims^2, 5^. However, certain metrics may be more sensitive to identifying change in particular contexts^29^. For example, we found large differences in functional evenness between the total catch and reported catch for the Desventuradas Islands of Chile. However, reported catch is only based on three taxa and is predominantly comprised of a single species (Chilean Jack Mackerel), giving a low functional evenness for reported catch. As a result, including unreported catches where reported fisheries rely on a small number of target species may have a larger impact on evenness than in a diverse reported fishery even if the proportion of unreported catch is low. Similarly, we focused on metrics that account for the biomass of exploited taxa. Functional metrics based on presence-absence data, such as functional richness, may be more sensitive to identifying changes in the exploitation of comparatively rare functions among different catch groupings^29^. Such an approach would be important if rare functions are identified as being particularly vulnerable to fisheries exploitation, and are central to ecosystem function and resilience^7, 30^. For example, a few browsing herbivores are critical for reversing coral to algal regime shifts on coral reefs, yet browsers are particularly vulnerable to fishing^30^.

Additionally, metric selection may depend on how well unreported catches within an EEZ can be assessed, specifically with respect to the certainty assigned to a reconstruction. The estimated accuracy of the catch reconstruction varies spatially^13, 22^; our findings suggest that where there are difficulties in reconstructing unreported catches, certain functional metrics may be less influenced. Specifically, we found that differences between total and reported functional dispersion was generally lower across EEZs than differences in evenness, such that accounting for unreported catch may be less critical when exploring trends in dispersion than evenness (Recommendation 2, Table 2). Thus, investigation into the range of functional roles present in the catch may be possible even where reconstruction of unreported catches is challenging, while evaluation of the relative dominance of particular functions may be less robust in this context.

Research exploring patterns in fisheries functional diversity and traits is an expanding literature that exploits broad-scale, comprehensive datasets. However, whether these trends reflect corresponding shifts in the functional composition of fish communities needs to be assessed^16, 31^. What fishes are caught is not only driven by their abundance within the fish community, but is also related to gear selectivity and fishing capacity, targeting capabilities, cultural norms and dietary preferences, regulations, and market conditions^16, 23, 32, 33^. Furthermore, these factors vary regionally and change over time, and may amplify or dampen the underlying community trends. The functional diversity of fisheries is an important metric in itself, for example, work by Dee *et al*. highlights functional diversity may buffer the negative impacts of temperature variability in relation to fisheries’ yields, thus providing an indication of economic risk and stability^9^. Nonetheless, understanding the links between functional diversity trends in the catch and in the community is essential to make the connections between patterns in fisheries exploitation and ecosystem function and resilience over time. Future studies need to compare functional diversity trends in fisheries-dependent and independent datasets to tease apart fishing, habitat and environmental effects on functional diversity patterns^34^ (Recommendation 3, Table 2), and move beyond the understanding of fisheries reporting effects described here. Such work will provide an understanding of the broad-scale temporal and spatial drivers of change in functional diversity in both fisheries and fish communities.

Exploring spatial variation in the proportion of catch within different groupings was not a core focus of our study. Nonetheless, these findings provide an interesting perspective on the contribution of different catch groupings in space. Low reporting and limited high certainty data were not characteristics of specific latitudes or oceans, and do not correspond to regions with few stock assessments^35^. This contrasts with patterns of coverage in biodiversity datasets on land, where there are strong regional patterns^36^. Furthermore, although small-scale fisheries may be particularly critical for food security in the global south^37^, there is no apparent latitudinal or socioeconomic gradient in the proportion of the catch arising from small-scale fisheries. Understanding these patterns, gaps in reporting, and the distribution of specific sectors is critical to be able to explore the potential impact on indicators developed from these data. This knowledge also supports more effective and targeted data collection aimed at improving data coverage and thus indicator efficacy^36^.

Quantitative metrics are important for assessing progress against management objectives, whether fisheries or conservation oriented. The use of clear metrics is important to regional management but also to track performance against global targets such as the Aichi biodiversity targets or the Sustainable Development Goals^11^. Certainly, the increasing interest in exploring functional diversity trends^4^, and the extensive temporal and spatial coverage of the SAUP data makes it an attractive data source for quantifying function specific indicators. Nevertheless, although we found little impact on global trends of functional diversity of accounting for unreported catches, this outcome does not account for the impact of the recording and reconstruction process on derived functional diversity estimates. Each stage in the reporting and reconstruction process affects the taxonomic resolution of fisheries data. The process by which different nations report catches to the FAO does not promote recording of data at a fine taxonomic resolution^13^. Furthermore, there are potential biases in the FAO data, such as an increase in the diversity of reported taxa over time (Supplementary Fig. S8). Although this trend may be driven in part by changing fishing practices^17^, changes in species identification and reporting may also be a contributing factor. SAUP makes significant effort to counteract these issues by sourcing missing data^13^. In supplementing the reported data with information from other sources, the SAUP process of catch reconstruction may increase the taxonomic resolution of the data, but often with highly variable uncertainty among countries. For example, for Angola the taxonomic composition of discards is based on data from Gabon^38, 39^ whereas for New Zealand, the literature provided information on 400 species that have been recorded as discards in different fisheries within the EEZ^40^. A key strength of the reconstructed catch data are their comprehensiveness. Nonetheless, taxonomic artefacts in the FAO data are overlain by methodological assumptions made at the reconstruction phase, such that considerable uncertainty remains regarding the taxonomic identity of certain components of the catch^12–14^. These uncertainties are highlighted on the SAUP website, where it recommends that only certain subsets of data are used for trophic or trait-based indicators, based on the degree of taxonomic pooling^13^. Nonetheless, much of the SAUP data has missing certainty data, which refers to the likely accuracy of the fisheries data^14^, making it difficult to assess how data quality influences functional metric estimates. Increasing the comprehensiveness of the certainty data is a clear research need (Recommendations 4&5, Table 2). It is now critical we explore how the process of reporting and reconstructing global catches influences our understanding of functional diversity trends Recommendations 6–10, Table 2). For example, simulation testing of reconstruction methods would allow us to explore the effects of this process on functional diversity trends.

In conclusion, we show that different components of the catch such as reported and unreported data, and industrial and small-scale sectors have different functional fingerprints. These differences may be discerned despite broad-scale variability in fishing practices, habitat and biophysical conditions. The impact of unreported catches on functional diversity patterns is scale-dependent, with larger impacts at local to regional scales than at a global scale. Critically, management decisions are made at these smaller scales, indicating that accounting for unreported fisheries in functional diversity patterns is fundamental to supporting fisheries management. In particular, where there is low certainty regarding unreported catches, this may have significant impacts on our understanding of the effects of fishing on functional diversity and the knock-on consequences for ecosystem structure, function and resilience

Selection of functional metrics depends on the research aims, but our findings suggest that functional dispersion may be less susceptible to missing data such as bycatch and illegal fishing, than functional evenness. Therefore, dispersion may provide a more robust metric in EEZs where reconstruction of unreported catches is proving challenging. Similarly, basing functional diversity analyses on either coarse functional groupings or fine-scale traits depends on the focus of the study, however, at the global scale, unreported catches has similar impacts on functional diversity trends regardless of the resolution of the function data. Exploring the influence of additional aspects of the catch reporting and reconstruction methods and assumptions on functional diversity trends was beyond the scope of this study. However, we provide a series of clear recommendations to build understanding in regards to the impact of the reconstruction process on functional diversity patterns. Increasing the comprehensiveness of the certainty data associated with the catch information is an important first step.

## Methods

### Catch data

The SAUP provides spatially explicit catch data at a range of taxonomic resolutions from species up to broad classes such as “marine fin fishes”^13^. SAUP categorises components of the catch according to reporting status, certainty score and fishing sector (Supplementary Table S1A). Reporting status refers to reporting of catches in official figures e.g. national and FAO databases (Fig. 1a). Unreported catches are estimated based on literature searches, expert opinion and local knowledge. Certainty scores relate to the estimated accuracy of the reconstructed catch data^13^ and is based on qualitative expert assessment of the relative accuracy of the catch estimate derived from the underlying data, assumptions and methods used for each sector and country, and was developed from a similar approach used in the IPCC process^22^. Catch data were downloaded from the SAUP website for each EEZ in 2010.

### Trait data

The functional roles of species may be described using broad functional groups, or using more detailed information on a range of life-history, ecological and behavioural traits^2, 5^. We explored functional trends using both approaches. Broad functional groups (e.g. Small pelagic) are provided within the SAUP data (Supplementary Table S2A). These groupings are available for all taxa, regardless of taxonomic resolution and thus functional metrics based on the functional groups were estimated using all the catch data. Fine-scale trait data were downloaded from FishBase using rFishBase^41, 42^. The six traits (habitat, minimum depth, maximum depth, trophic level, maximum length, and body shape) were selected to reflect the role of a species within the ecosystem (Supplementary Table S2B), and where data were readily available. For example, the minimum and maximum temperature range of a species may reflect where a species lives and feeds, but these data are poorly represented in FishBase^9^. In FishBase, trait data are presented at the species level. Where trait information was lacking at the species level, genus level averages were used (mean for continuous traits and mode for categorical traits)^16^. Where catch data were recorded at the genus level, the average values for species within that genus were used. Catch data recorded at a taxonomic resolution of family or lower were not incorporated into the estimate of functional diversity based on fine-scale trait data as averaged trait values at these lower resolutions would bias estimates of diversity. The proportion of catch in the SAUP database presented at species and genus level varied amongst EEZs (Supplementary Fig. S9). An underlying assumption of using a single estimate for each trait for a species is that trait values are fixed and do not change for a particular species in time, space or as individuals grow. Thus, spatial trends in functional metrics estimated from these traits are representative of shifts in the composition of the catch^16^. The SAUP catch data cover vertebrate and invertebrate species, but the majority of the global catch is composed of fishes (finfish and chondricthyans; Supplementary Fig. S10), and little trait data are available for invertebrate species (SeaLifeBase), therefore our analyses focused on fishes only.

### Functional diversity

Functional diversity may be represented by a variety of complementary and overlapping metrics^43^. We explored functional diversity by estimating functional evenness and functional dispersion using the FD package in R^44^. Functional evenness describes how evenly taxa are distributed in trait space (Fig. 1b). Functional dispersion is the distance of each taxon from the central point in trait space occupied by all the samples, and is strongly correlated with Rao’s Q, another metric commonly used to measure functional diversity^44^. Both metrics were weighted by biomass of each taxon, such that the estimates are influenced by the relative prevalence of each taxon in the catch and are not sensitive to outliers. As a result, the estimates were influenced little by trait combinations and functional groups that were rarely caught. Functional richness, a metric based on presence-absence data, and thus more sensitive to rare functions, was also estimated. However, the results are not presented here because this metric is estimated from a convex hull volume produced from the axes returned from a principle coordinates analysis (PCoA), and this reduced-space representation produced by the FD package^44^ was of low quality (quality of the representation is indicated by an R^2^ value).

The metrics were estimated using the Gower dissimilarity index, which allows for numeric, ordinal and categorical traits, and allows for some missing values. All traits were equally weighted. A Cailliez correction factor was used to account for negative eigenvalues in the distance matrix. The functional metrics were estimated for every EEZ in 2010, first using all the data, then for different components (termed ‘groupings’) of the catch in relation to reporting, uncertainty and sector (Supplementary Table S1B). These calculations were repeated using both the SAUP functional groups for the whole catch regardless of taxonomic resolution and the finer-scale traits for the catch identified to species or genus level (Supplementary Table S3).

### Data analysis

To understand spatial variability in the proportion of catch within different catch groupings, proportion data were mapped by EEZ. Spearman rank correlations were used to investigate the relationship between the proportion of catch in the different groupings, for example, the correlation between the proportion of catch reported and the proportion of the catch caught by the small-scale fishery sector.

Three steps were used to characterise the functional fingerprint of the different catch groupings and explore the relationship between functional metric estimates for catch data within the different groupings: (1) Functional estimates were mapped for each of the catch groupings; (2) spearman rank correlations were estimated among functional metric estimates for the catch groupings; and (3) Wilcoxon paired tests were used to evaluate differences in the distributions of functional metric estimates among the different groupings. To explore how the functional fingerprint of the different catch groupings influences the functional patterns of the total catch, spearman rank correlations were calculated between the catch groupings and the total catch. These analyses were repeated using functional estimates calculated from the functional group data and the fine-scale trait data.

Each EEZ had a different proportion of the catch within reported, high certainty and industrial groupings. To understand which EEZs show greater differences in the functional metrics between the total catch and specific catch groupings, the difference between total catch functional metric estimates and those for reported and industrial fishing were mapped.

### Data availability

All data used in the study are available for download from the Sea Around Us website (seaaroundus.org) and FishBase (fishbase.org).

## Electronic supplementary material

Supplementary Material
