# Supplementary material for: Improving understanding of the functional diversity of fisheries by exploring the influence of global catch reconstruction
Source: Sci Rep. 2017 Sep 6;7:10746. doi: 10.1038/s41598-017-10723-1 (PMC5587757; doi:10.1038/s41598-017-10723-1)
Supplement: Supplementary file 1 — Supplementary Material [file 41598_2017_10723_MOESM1_ESM.pdf]

Supplementary Material for

Improving understanding of the functional diversity of fisheries by exploring the influence of global catch reconstruction

Authors: Kirsty L. Nash, Reg A. Watson, Benjamin S. Halpern, Elizabeth A. Fulton, Julia L. Blanchard

Supplementary Note 1: Supplementary results – proportion of catch in different catch groupings

Supplementary Note 2: Supplementary results – correlations among proportions in different catch groupings

Figure S1: Spatial variation in functional evenness for different catch groupings, where analyses based on functional groups

Figure S2: Spatial variation in functional dispersion for different catch groupings, where analyses based on functional groups

Figure S3: Spatial variation in proportion of catch in different catch groupings by EEZ for catch identified to species and genus level

Figure S4: Spatial variation in proportion of catch in different catch groupings by EEZ for total catch regardless of taxonomic resolution

Figure S5: Correlations among proportions of catch in different catch groupings for catch identified to species and genus level

Figure S6: Correlations among proportions of catch in different catch groupings for total catch regardless of taxonomic resolution

Figure S7: Proportion of catch within different catch grouping for each EEZ.

Figure S8: Change in taxonomic richness in FAO data 2050-2014

Figure S9: Proportion of catch at species and genus level resolution

Figure S10: Proportion of fish in catch

Table S1: Catch groupings within fisheries data

Table S2: Trait and functional group data

Table S3: Functional diversity metrics

Supplementary Note 1: Results of analyses exploring spatial distribution in the proportion of data in different catch groupings.

There is geographic variation among EEZs in the proportions of catch within catch groupings (Fig. S3&S4). However, there does not appear to be clear latitudinal, socioeconomic or ocean-specific patterns in this variation, for example both high and low latitude EEZs exhibit either high or low proportions of reported and industrial catch. Most EEZs had high certainty classifications assigned to relatively small proportions of the catch (pale shading in Fig. S3C & Fig. S4C). Variation among EEZs appear similar when focusing on the section of the catch identified to species or genus level or on the whole catch regardless of taxonomic resolution (Fig. S3 vs. Fig. S4, e.g. Fig. S3A vs. Fig. S4A).

There was weak or no correlation between either functional evenness (trait analysis: spearman rho=0.04, functional group analysis: spearman rho=-0.27) or functional dispersion (trait analysis: spearman rho=-0.27, functional group analysis: spearman rho=-0.31) and the proportion of catch reported. Similarly, there was no correlation between either functional evenness (trait analysis: spearman rho=0.00, functional group analysis: spearman rho=-0.23) or functional dispersion (trait analysis: spearman rho=-0.17, functional group analysis: spearman rho=-0.15) and the proportion of industrial catch.

Supplementary Note 2: Results of analyses exploring correlations among proportions in catch groupings.

Proportion of reported catch data was moderately positively correlated with proportion of catch arising from industrial fisheries (Fig. S5; Spearman rank rho = 0.48 to 0.54) and moderately negatively correlated with small-scale fisheries (Spearman rank rho = -0.41 to -0.48). Thus, the greater the proportion of the catch from small-scale fisheries, the smaller proportion of the catch tends to be reported within an EEZ. Unexpectedly, there was no relationship between reported catch and high certainty data (Fig. S5, Spearman rank rho = -0.086 to 0.012). The proportion of the catch reported was moderately negatively correlated with the proportion of low certainty data, and was moderately positively correlated with the proportion of missing certainty data. Thus, as more of the catch is reported, the proportion of low certainty data decreases, but the proportion of high certainty data does not increase. The lack of a relationship between reported and high certainty data is likely driven by the large amount of missing data on certainty classifications, and suggests that trends arising from level of certainty are likely to be misleading. As a result, certainty classifications were not used in the functional diversity analyses.

Note, catch is either reported or unreported, therefore those catch groupings that are positively correlated with reported data are negatively correlated with unreported data. In contrast, catch data are either from industrial, small-scale or recreational fisheries. Therefore, the correlations between industrial fisheries and reported data are not the exact negation of the correlations between small-scale and reported data.

FIGURE S1: Spatial variation in functional evenness. Functional evenness in Ai) reported, Aii) unreported, Bi) industrial and Bii) small-scale catches for EEZs in 2010. Estimation of functional evenness based on functional group data and the whole catch regardless of taxonomic resolution. Grey shading in EEZs represents missing data, where there were either too few functionally distinct taxa to estimate evenness or insufficient data at the species/genus level (trait analysis only). Maps created in using the ggplot2[1] package in R[2].

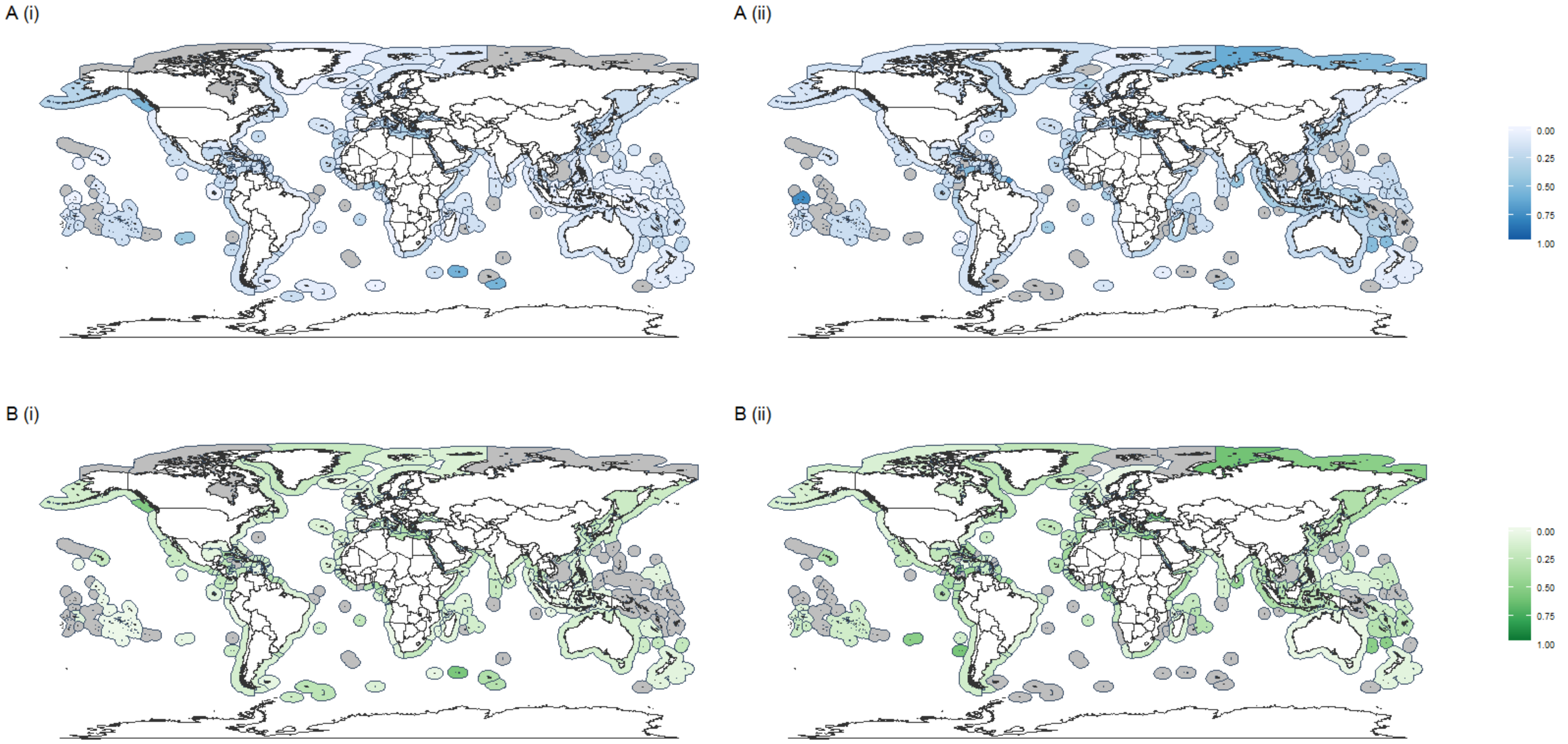

FIGURE S2: Spatial variation in functional dispersion. Functional evenness in Ai) reported, Aii) unreported, Bi) industrial and Bii) small-scale catches for EEZs in 2010. Estimation of functional evenness based on functional group data and the whole catch regardless of taxonomic resolution. Grey shading in EEZs represents missing data, where there were either too few functionally distinct taxa to estimate evenness or insufficient data at the species/genus level (trait analysis only). Maps created in using the ggplot2[1] package in R[2].

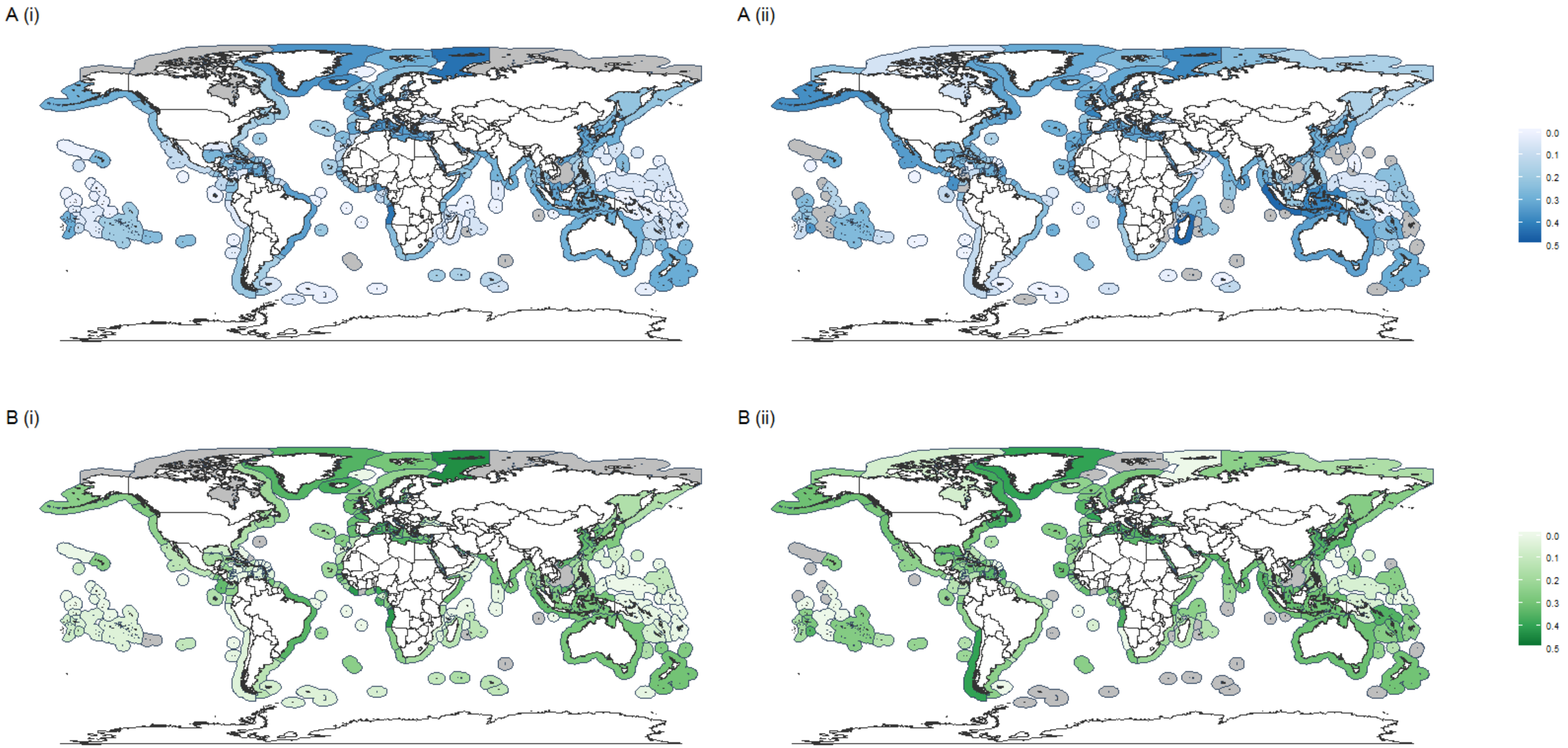

FIGURE S3: Proportion of catch identified to species or genus level within different EEZs in 2010 from A) reported, B) industrial, and C) high certainty data. Maps created in using the ggplot2[1] package in R[2].

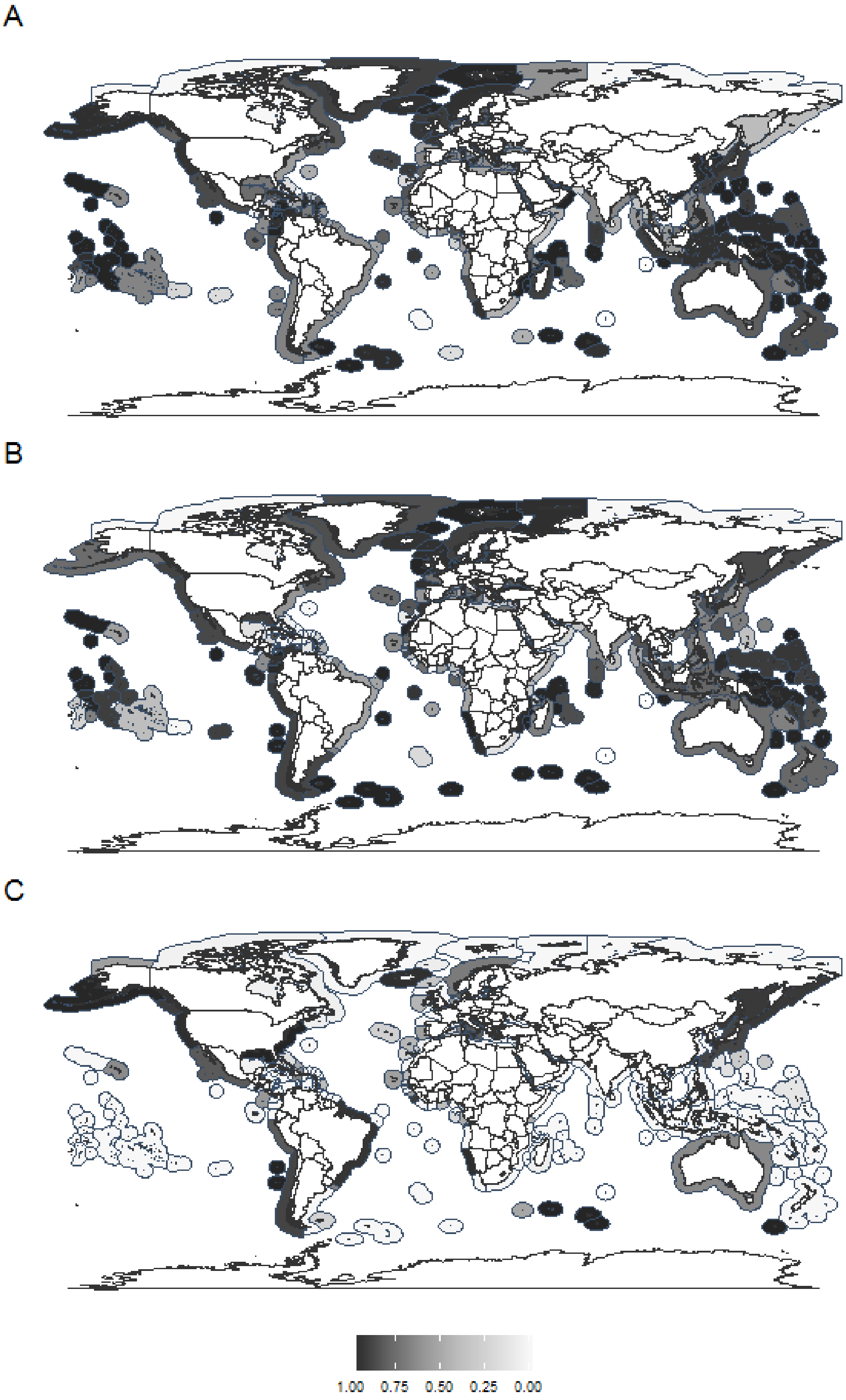

FIGURE S4: Proportion of catch within different EEZs in 2010 from A) reported, B) industrial, and C) high certainty data. Catch covers all taxa, regardless of the taxonomic resolution at which they were identified. Maps created in using the ggplot2[1] package in R[2].

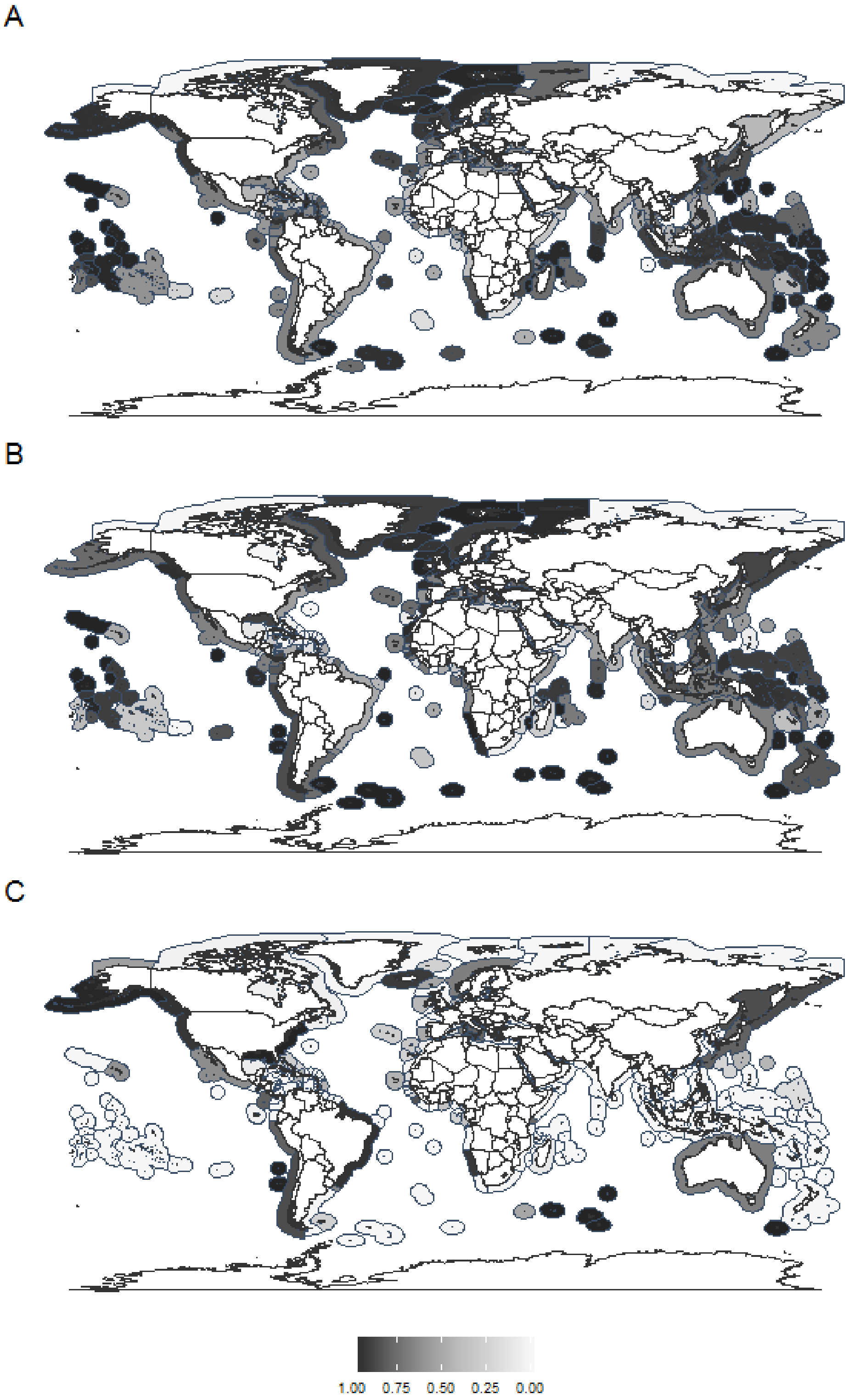

FIGURE S5: Spearman rank correlations among the proportion of catch (tonnes) in different catch groupings for catch recorded at species or genus level. Proportion of unreported data are not shown on the plot because all catch are assigned either reported or unreported status, therefore correlations between unreported data and the other catch groupings are the negation of those for reported data. Prop_UNA represents missing certainty data.

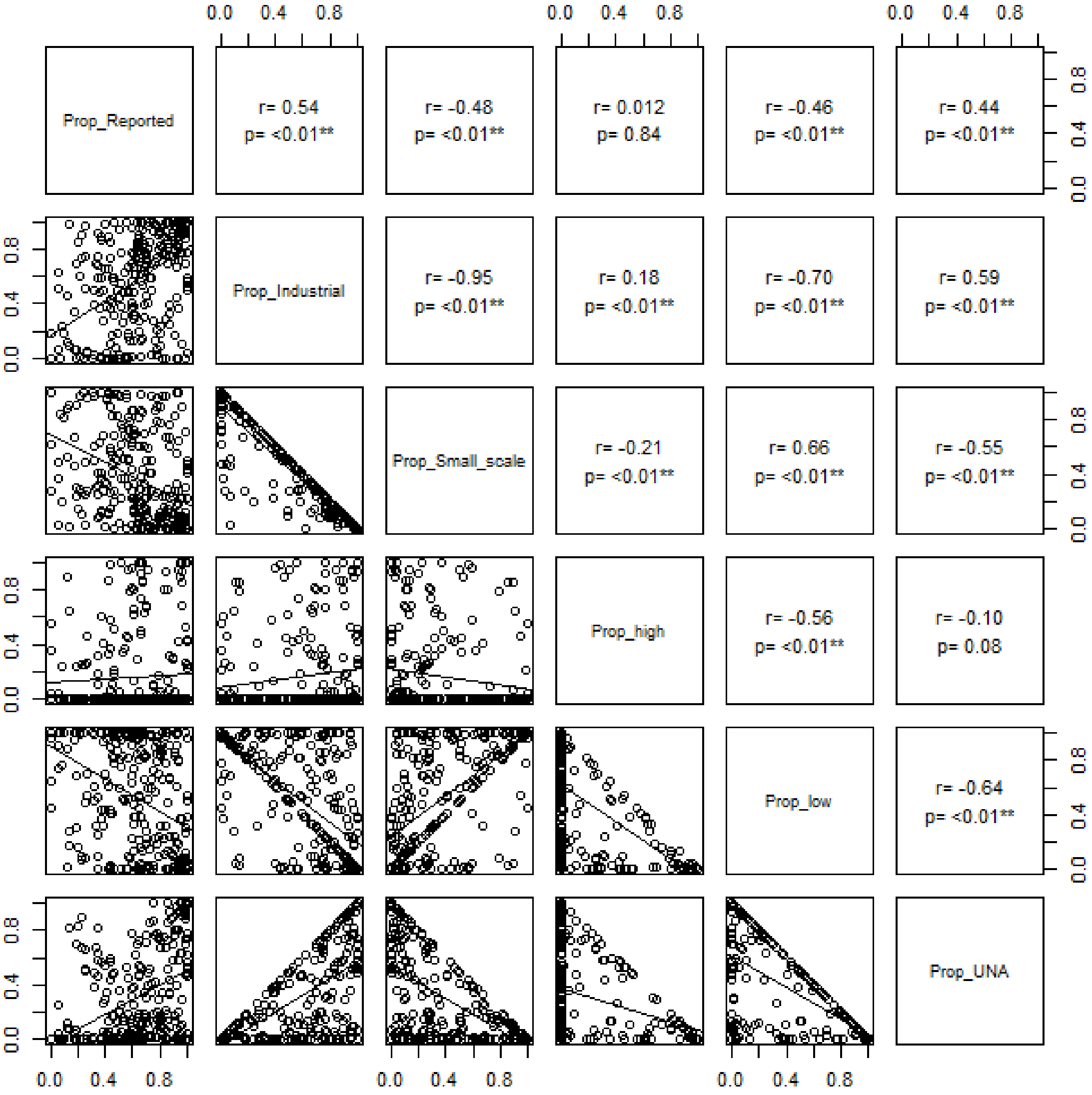

FIGURE S6: Spearman rank correlations among the proportion of catch (tonnes) in different catch groupings for the whole catch regardless of taxonomic resolution. Proportion of unreported data are not shown on the plot because all catch are assigned either reported or unreported status, therefore correlations between unreported data and the other catch groupings are the negation of those for reported data. Prop_UNA represents missing certainty data.

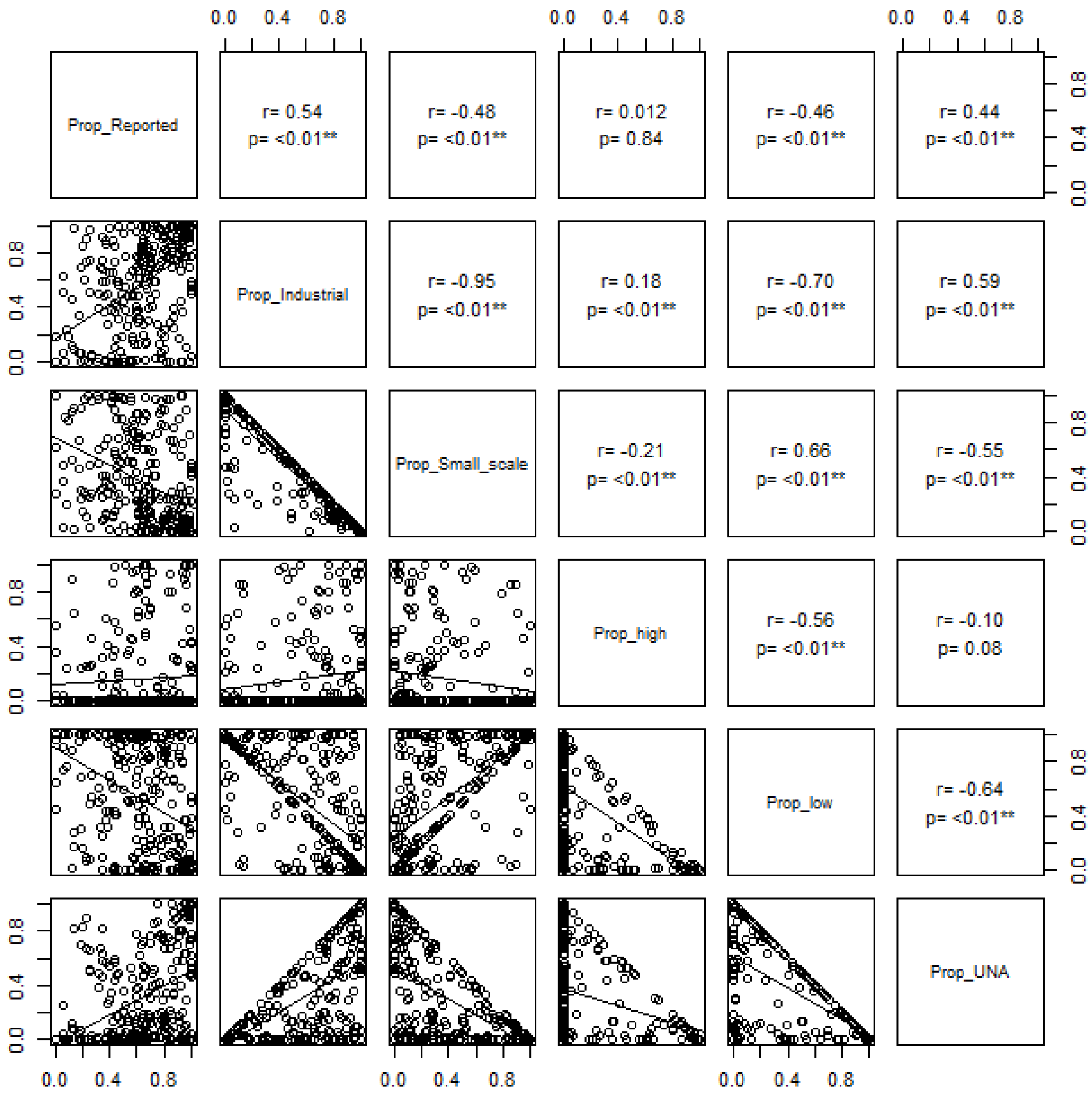

FIGURE S7: Proportion of catch within different EEZs A) reported and B) from the industrial sector. Proportions are estimated for (i) the whole catch regardless of taxonomic resolution and (ii) for catch identified to the species and genus level.

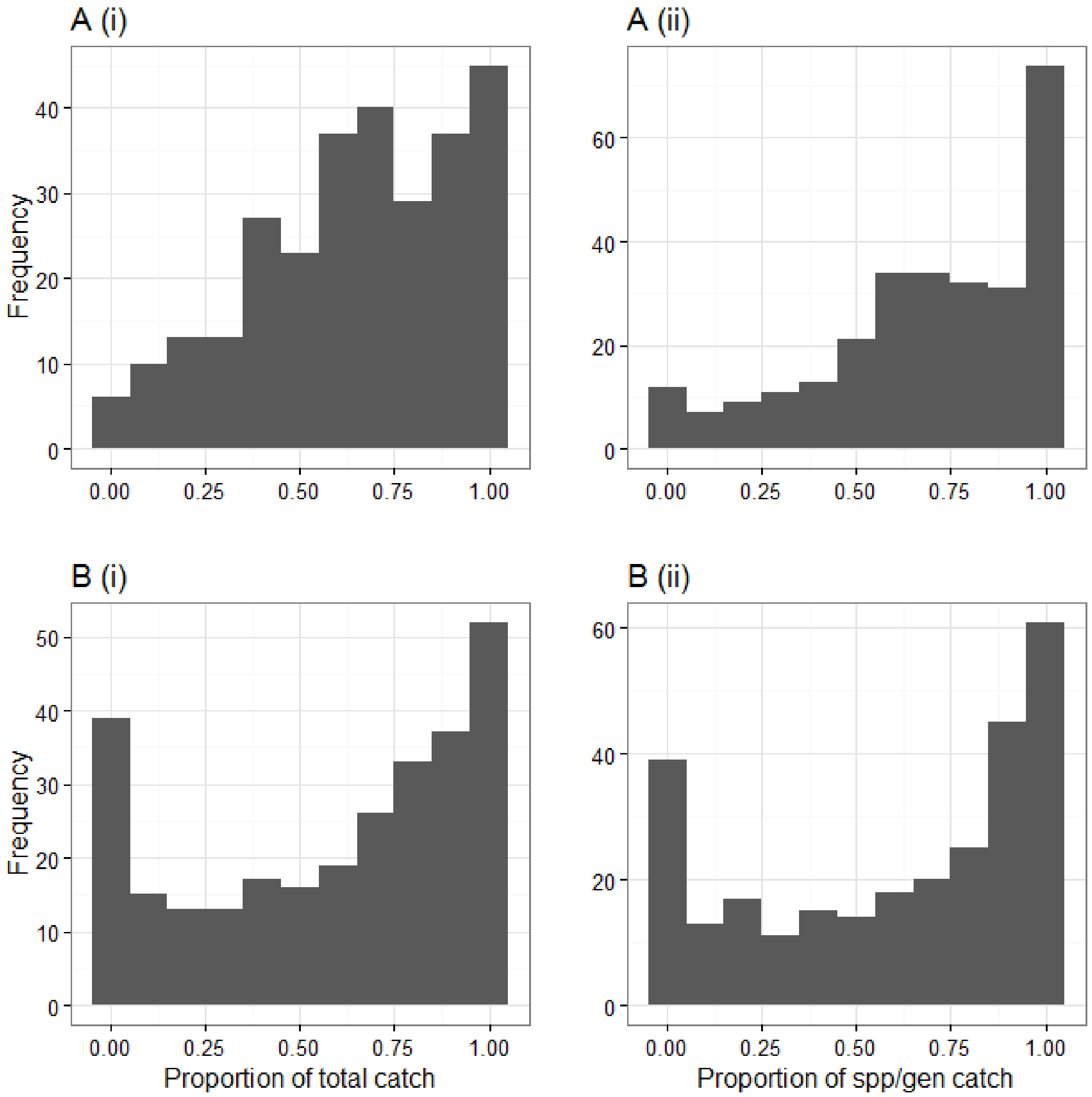

FIGURE S8: Change in taxonomic richness within FAO database 1950 to 2014.

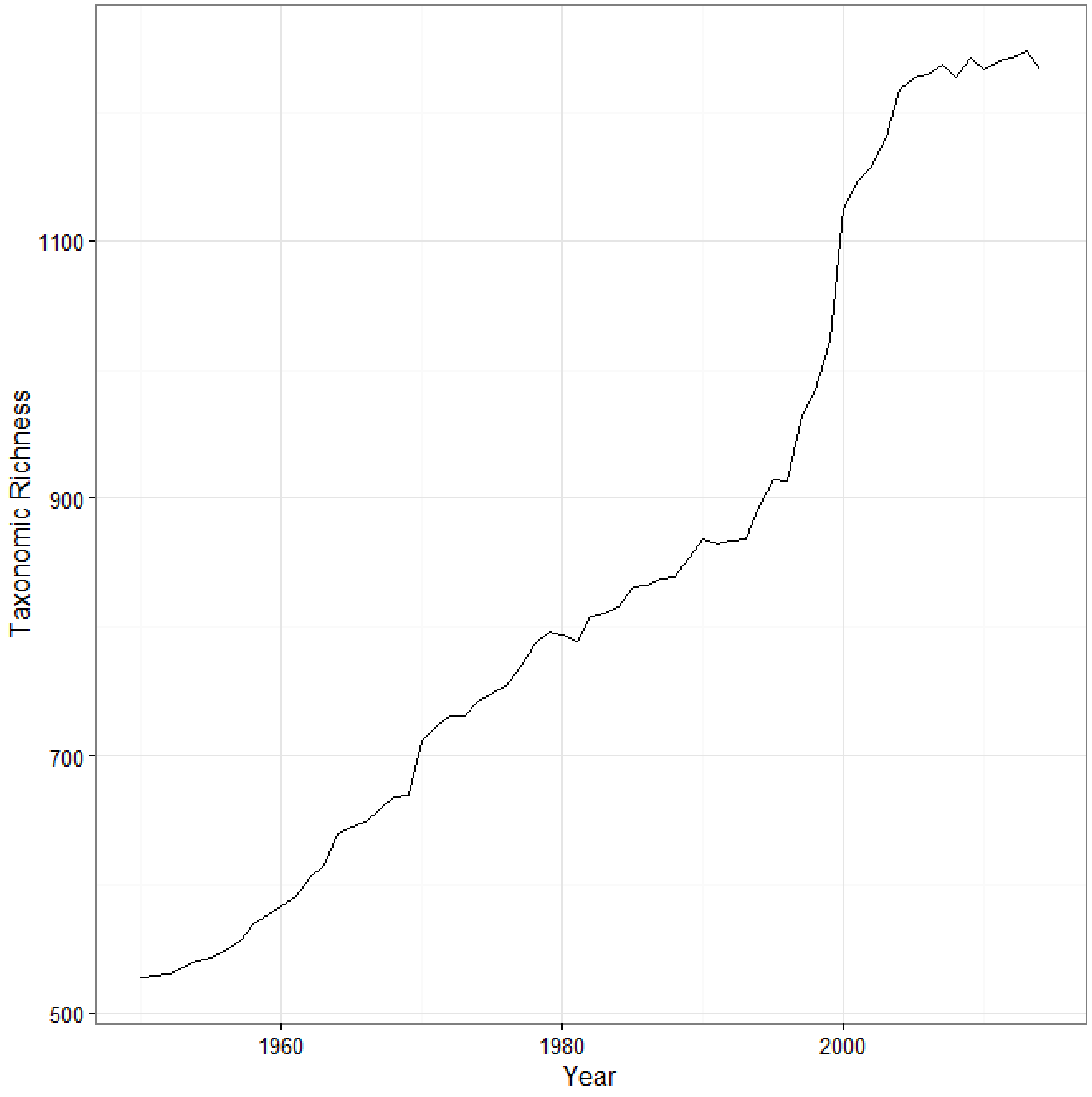

FIGURE S9: Proportion of catch data from each EEZ at the species or genus level taxonomic resolution.

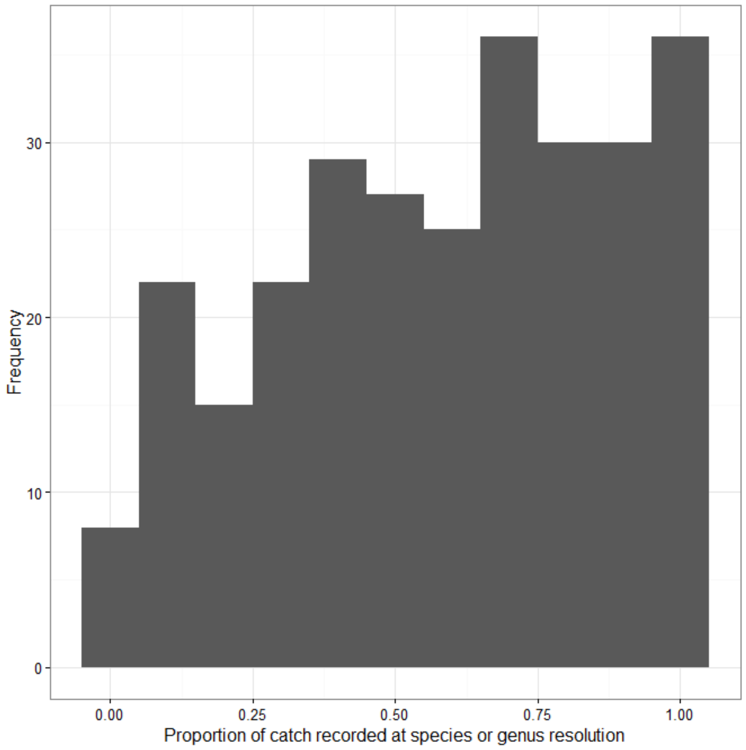

FIGURE S10: Proportion of fish in catch data for each EEZ. Bahamas, Canada (East Coast), Germany (North Sea), Honduras (Caribbean), Nicaragua (Pacific), South Georgia & Sandwich Islands, South Orkney Islands, Tristan de Cunha Islands and USA (East Coast) were the locations where the proportion of fish was below 0.5.

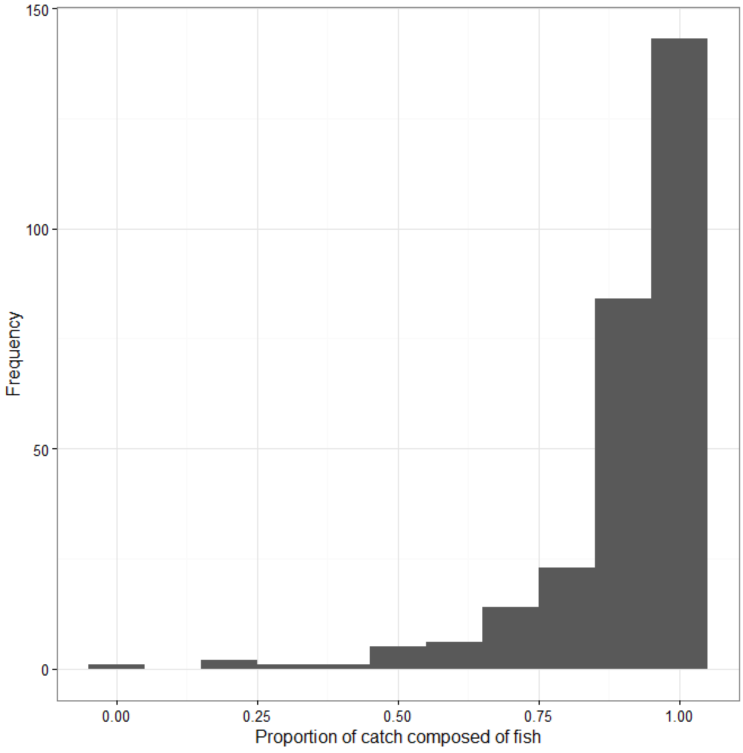

TABLE S1: Categorisation of catch data in A) Sea Around Us database and B) as used in functional diversity estimates.

Reporting status	Uncertainty score	Sector
**A) Sea Around Us database**		
Reported	Very low certainty (U1)	Industrial
Unreported	Low certainty (U2)	Recreational
	High certainty (U3)	Subsistence
	Very high certainty (U4)	Artisanal
	No score assigned (UNA)	
**B) Functional diversity estimates**		
Reported	Low certainty (U1&U2)	Industrial
Unreported	High certainty (U3&U4)	Small-scale (Subsistence & Artisanal)
	No score assigned (UNA)	

TABLE S2: Traits incorporated into functional diversity estimates, based on A) SAU broad functional group, and B) finer scale traits. The Sea Around Us functional group were split into two separate traits (group and size) when estimating the functional diversity metrics.

**A) Sea Around Us functional groups**
Small bathydemersal (<30 cm)
Medium bathydemersal (30 - 89 cm)
Large bathydemersal (>=90 cm)
Small bathypelagic (<30 cm)
Medium bathypelagic (30 - 89 cm)
Large bathypelagic (>=90 cm)
Small benthopelagic (<30 cm)
Medium benthopelagic (30 - 89 cm)
Large benthopelagic (>=90 cm)
Small demersal (<30 cm)
Medium demersal (30 - 89 cm)
Large demersal (>=90 cm)
Small pelagic (<30 cm)
Medium pelagic (30 - 89 cm)
Large pelagic (>=90 cm)
Small reef assoc. fish (<30 cm)
Medium reef assoc. fish (30 - 89 cm)
Large reef assoc. fish (>=90 cm)
Small to medium flatfishes (<90 cm)
Large flatfishes (>=90 cm)
Small to medium rays (<90 cm)
Large rays (>=90 cm)
Small to medium sharks (<90 cm)
Large sharks (>=90 cm)

**B) Fine-scale traits**		
Trait	Description	Relevance
Habitat	Categorical variable: benthic, demersal, bathydemersal, bathypelagic, benthopelagic, reef-associated, pelagic-neritic, pelagic-oceanic	Where species providing role.
Minimum Depth	Ordinal variable: <10, 10-29.9m, 30-49.9m, 50-99.9m, 100-199.9m, 200-499.9m, 500-999m, >=1000m	Where species providing role. Also correlated to temperature requirements [3].
Maximum Depth	Ordinal variable: <10, 10-29.9m, 30-49.9m, 50-99.9m, 100-199.9m, 200-499.9m, 500-999m, >=1000m	Where species providing role. Also correlated to temperature requirements [3].
Trophic Level	Continuous variable	What species are eating
Maximum Length	Continuous variable	Size correlated with size of prey and a range of life-history traits [4].

Body Shape	Categorical variable: eel-like, elongated, fusiform, short/deep, flat	Type of environment likely to be living in e.g. high or low water movement; predation mode e.g. ambush predator [5].

TABLE S3: Functional diversity calculations used in the study.

**Traits**	**Catch groupings**	**Catch data**	**Functional metrics**
SAU functional groups	Total catch<br>*Reporting status*<br>• Reported<br>• Unreported<br>*Certainty score*<br>• Low certainty<br>• High certainty<br>*Sector*<br>• Industrial<br>• Small-scale	Whole catch dataset, regardless of taxonomic resolution	• Functional evenness<br>• Functional dispersion
Fine-scale traits	Total catch<br>*Reporting status*<br>• Reported<br>• Unreported<br>*Certainty score*<br>• Low certainty<br>• High certainty<br>*Sector*<br>• Industrial<br>• Small-scale	Catch data identified to species and genus resolution	• Functional evenness<br>• Functional dispersion

References

1. Wickham H. *ggplot2: elegant graphics for data analysis*. Springer-Verlag (2009).

2. R Core Team. R: a Language and Environment for Statistical Computing. R Foundation for Statistical Computing, Vienna, Austria. https://www.R-project.org (2016).

3. Dee LE, *et al.* Functional diversity of catch mitigates negative effects of temperature variability on fisheries yields. *Proc Royal Soc B* **283**, (2016).

4. Abesamis RA, Green AL, Russ GR, Jadloc CRL. The intrinsic vulnerability to fishing of coral reef fishes and their differential recovery in fishery closures. *Rev Fish Biol Fisheries* **24**, 1033-1063 (2014).

5. Tytell ED, Borazjani I, Sotiropoulos F, Baker TV, Anderson EJ, Lauder GV. Disentangling the functional roles of morphology and motion in the swimming of fish. *Integrative and Comparative Biology* **50**, 1140-1154 (2010).
